# Dual-lumen catheters for continuous venovenous hemofiltration: limits for blood delivery via femoral vein access and a potential alternative in an experimental setting in anesthetized pigs

**DOI:** 10.1186/cc5693

**Published:** 2007-02-15

**Authors:** Juliane K Unger, Klaus Pietzner, Roland C Francis, Juergen Birnbaum, Marc Michael Theisen, Arne-Joern Lemke, Stefan M Niehues

**Affiliations:** 1 Department of Comparative Medicine and Laboratory Animal Sciences, Charité Campus Virchow-Klinikum, Universitätsmedizin Berlin, Augustenburger Platz 1, D-13353 Berlin, Germany; 2 Department of Anesthesiology and Intensive Care Medicine, Charité Campus Virchow-Klinikum, Universitätsmedizin Berlin, Augustenburger Platz 1, D-13353 Berlin, Germany; 3 Department of Anesthesiology and Intensive Care Medicine, Charité Campus Mitte, Universitätsmedizin Berlin, Charitéplatz 1, D-10117 Berlin, Germany; 4 Department of Anesthesiology and Intensive Care, University Hospital, Albert-Schweitzer-Str. 33, D-48149 Muenster, Germany; 5 Department of Radiology, Charité Campus Virchow-Klinikum, Universitätsmedizin Berlin, Augustenburger Platz 1, D-13353 Berlin, Germany

## Abstract

**Introduction:**

Small intravascular volume, pathophysiological hemorheology, and/or low cardiac output [CO] are assumed to reduce available blood flow rates via common dual-lumen catheters (except for those with a right atrium catheter tip position) in the critically ill patient. We performed an experimental animal study to verify these assumptions.

**Methods:**

Anesthetized, ventilated pigs (35 to 50 kg) were allocated to different hemorheological conditions based on the application of different volume substitutes (that is, colloids and crystalloids, *n *= 6 to 7 per volume substitute). In a second step, allocation to the final study group was performed after the determination of the highest values for access flow (Qa) via an axial dual-lumen catheter (11 French, 20 cm long, side holes) placed in the femoral vein. High Qa rates (>300 ml/minute) were allocated to the dual-lumen catheter group; low Qa rates were switched to a 'dual-vein approach' using an alternative catheter (8.5-French sheath) for separate blood delivery. Hemodynamics (CO and central venous pressure [CVP]) and blood composition (blood cell counts, plasma proteins, and colloid osmotic pressure) were measured. Catheter tip positions and vessel diameters were exemplified by computed tomography.

**Results:**

Forty-four percent of the animals required an alternative vascular access due to only minimal Qa via the dual-lumen catheter. Neither hemorheologically relevant aspects nor CO and CVP correlated with the Qa achievable via the femoral vein access. Even though the catheter tip of the alternative catheter provided common iliac vein but not caval vein access, this catheter type enabled higher Qa than the dual-lumen catheter positioned in the caval vein.

**Conclusion:**

With respect to the femoral vein approach, none of the commonly assumed reasons for limited Qa via the arterial line of an axial dual-lumen catheter could be confirmed. The 8.5-French sheath, though not engineered for that purpose, performed quite well as an alternative catheter. Thus, in patients lacking right jugular vein access with tip positioning of large-French dual-lumen catheters in the right atrium, it would be of interest to obtain clinical data re-evaluating the 'dual-vein approach' with separate blood delivery via a tip-hole catheter in order to provide high-volume hemofiltration.

## Introduction

Dual-lumen catheters, genuinely engineered for vascular access in dialysis patients, are used for continuous venovenous hemofiltration (CVVH) in critically ill patients without affecting potentially different requirements such as for thrombogenecity, flow resistance, or hemodynamics. Baldwin and colleagues [1] described a mismatch of real blood flow (blood flow [Qb]) achieved via the arterial line of dual-lumen catheters and the Qb assumed to be achieved by the blood pumps. They found that the length of the filter life was negatively correlated with the percentage of Qb reduction by the pumps. Usually, in patients in whom the achievable Qb rates for renal replacement therapies are low, hemodynamics and hemorheology are severely deteriorated. Both aspects are assumed to affect the magnitude of negative pressure values arising from flow resistance via the catheter and in turn for the access flow (Qa) available during CVVH. The right jugular vein approach, including the catheter tip positioning in the right atrium [2], needs radiological control and very strict policies with respect to thrombogenecity and infections [3] but provides a Qa of 300 to 400 ml/minute. For various reasons, the right internal jugular vein approach is not feasible in all patients [4] and low Qb rates may become the main reason for short filter running times and limited clearance in crossflow-based apheresis filters [1,5-9]. Therefore, we performed a systematic experimental study in anesthetized, ventilated pigs to assess the commonly assumed correlation between the achievable Qb (achievable Qa) via the arterial line of a dual-lumen catheter placed in the femoral vein and the underlying hemodynamics (that is, cardiac output [CO] and central venous pressure [CVP]), catheter tip position, and hemorheological features (blood composition and volume substitute).

## Materials and methods

### Study design

**Figure 1:**
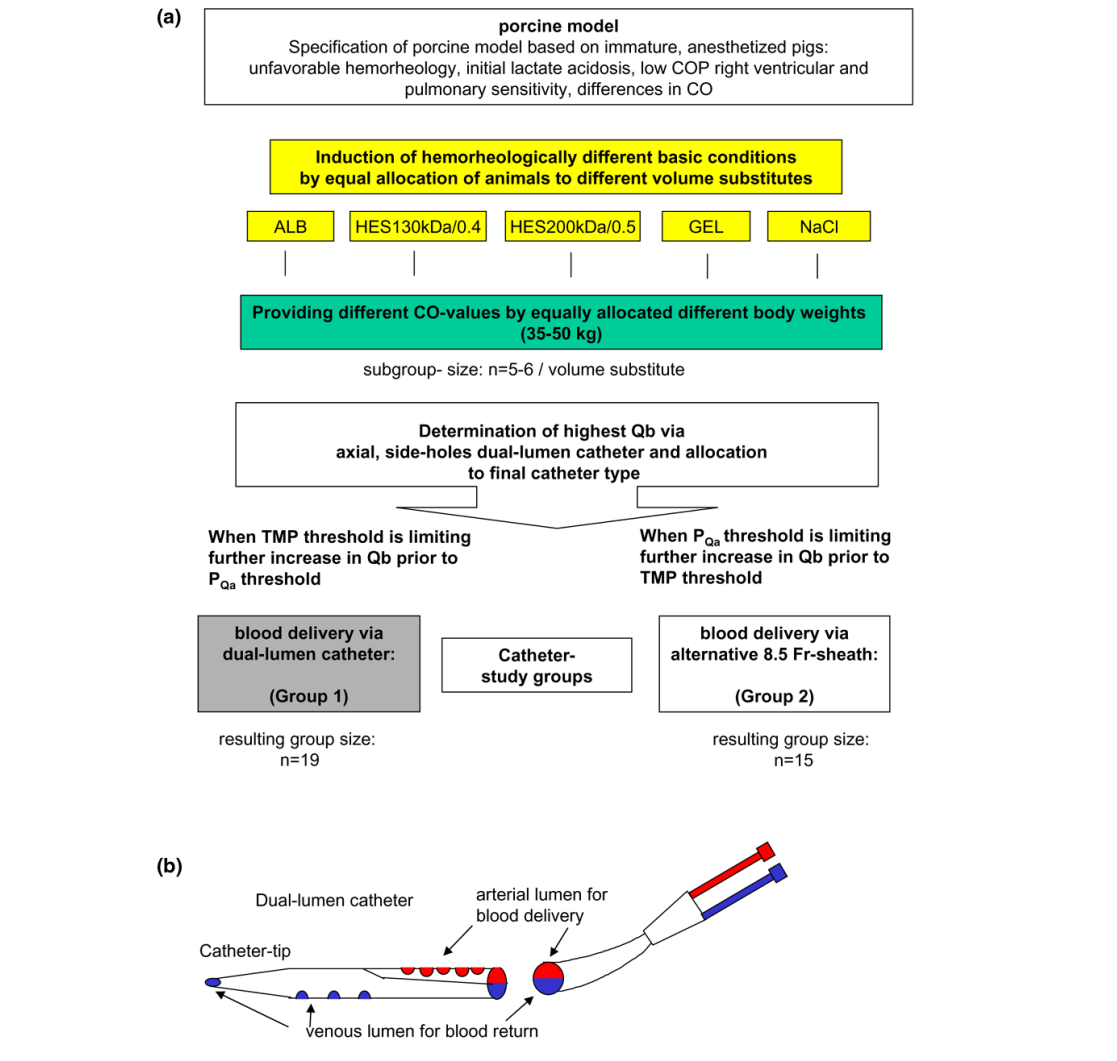
Assignment of animals to different basic conditions and catheter groups. **(a) **This schematic diagram shows the steps taken to form a clinically relevant range of hemorheological and hemodynamic conditions. Immature pigs provide a wide range of conditions because of their species-related physiological development and growth characteristics. Induction of anesthesia leads to transient lactate acidosis and increased lactate levels in the blood for up to several hours. Volume substitutes were chosen to correspond to the most commonly used and most controversial solutions. **(b) **Diagram of the type of dual-lumen catheter used in this study. ALB, human albumin; CO, cardiac output; COP, colloid osmotic pressure; GEL, gelatin; HES, hydroxyethyl starch; NaCl, normal saline; P_Qa_, pressure of access flow; Qb, blood flow; TMP, transmembrane pressure.

The study design, including the assignment of animals to a respective group, is explained in Figure 1a. In one group, an axial dual-lumen catheter (GamCath^®^, a polyurethane, 11-French, 20-cm-long, radiopaque catheter with blood return via a tip and three side holes in longitudinal line and blood delivery via five opposite side holes, as shown in Figure 1b; Gambro Dialysatoren GmbH, Hechingen, Germany) was used to operate CVVH. In case of low-flow problems, an alternative catheter (Alt Cath) (venous, single-lumen, polyurethane, 8.5-French sheath, 10-cm-long, radiopaque catheter chosen based on explorative *in vitro *evaluation; Arrow Deutschland GmbH, Erding, Germany) was used. Immature pigs were used to provide a wide range of different hemorheology patterns, CO values, and blood vessel diameters, as found in intensive care patients [6,8,9]. Furthermore, differences in volume management were investigated by using the most common solutions, which because of their rheological and anticoagulatory impact have been discussed for years. A total of 34 pigs were randomly assigned to fluid therapy with normal saline, 6% hydroxyethyl starch at 130 kDa/0.4 degrees of substitution, 6% hydroxyethyl starch at 200 kDa/0.05 degrees of substitution (all from Fresenius Kabi AG, Bad Homburg, Germany), albumin (ALB) (human albumin 20% diluted to 4% with normal saline; Baxter Deutschland GmbH, München-Unterschleißheim, Germany), or gelatin polysuccinat (Gelafundin^®^; B. Braun Melsungen AG, Melsungen, Germany). Ranges in CO, blood vessel diameters, and basic blood/plasma composition were achieved based on equally distributed differences of body weight (BW) from 35 to 50 kg.

### Identification of animals showing low flow rates via the dual-lumen catheter

All animals were placed in the supine position. After instrumentation of the animals, measurements of all hemodynamic and blood/plasma parameters for 'native' baseline (BS) were performed to ensure comparable basic conditions for the experiment. After bolus infusion of the respective volume substitute, the CVVH was started (running seven hours in total) using both lines of the inserted dual-lumen catheter, arterial and venous. The highest Qb rates achievable were determined for each animal, and accordingly they were allocated to one of the catheter groups, dual-lumen (group 1) versus Alt Cath (group 2) (Figure 1a). Because we had to consider potential hemolysis during high Qb rates within the hemofilter, we chose three thresholds that limited increases in Qb: (a) catheter type-related negative pressure arising from blood delivery via the arterial line of the respective vein access (pressure of Qa [P_Qa_] of less than -220 mm Hg), (b) the flow resistance in the venous line of the system (venous pressure [Pv] of more than 420 mm Hg), and (c) transmembrane pressure (TMP) of more than 180 mm Hg. In 19 out of 34 animals, the negative pressure for Qa could be tolerated (group 1). In contrast, in 15 animals, it was not possible to achieve similarly high flow rates without exceeding a P_Qa _of -220 mm Hg. This could not be improved by catheter rinsing, rotation, or tolerable forward/backward movement of the catheter or exchange of arterial to venous line and *vice versa*, and changes in the limb/pelvis positioning did not improve P_Qa_. In these animals, blood delivery was changed from the arterial line of the dual-lumen catheter to the Alt Cath (group 2), whereas blood return remained via the venous line of the dual-lumen catheter.

### Basic methods

#### Experimental animals

Female crossbred pigs were used (German Landrace × Large White, *n *= 34, weighing 40 ± 5 kg [mean ± standard deviation]). The study protocol was approved by the university animal care committee and the federal authorities for animal research in Berlin, Germany. The experiments were performed at the Department of Comparative Medicine and Laboratory Animal Sciences (certified by ISO [International Organization for Standardization] 9001). The principles of laboratory animal care were followed with respect to the guidelines of the European and German societies of laboratory animal sciences.

#### Anesthesia

Anesthesia was administered according to the following intravenous anesthesia regimen: Premedication consisted of intramuscular injection of azaperon (5 mg/kg Stressnil^®^; Janssen-Cilag GmbH, Neuss, Germany), ketamine (10 mg/kg Ursotamin^®^; Serumwerk Bernburg AG, Bernberg, Germany), and atropine sulfate (0.03 mg/kg Atropin Sulfat^®^; B. Braun Melsungen AG). Propofol injection enabled tolerance for intubation (intravenous 5 to 7 mg/kg Propofol 1% MCT^®^; Fresenius Kabi AG). Anesthesia was maintained by constant infusion of thiopentone (14 to 20 mg/kg per hour Trapanal^®^; ALTANA Pharma AG, now part of the Nycomed Group, Roskilde, Denmark) and fentanyl (3.5 to 6 μg/kg per hour Fentanyl^®^; Janssen-Cilag GmbH). Guidelines established for the determination of minimal anesthetic drug concentration in pigs were used to assess adequate depth of anesthesia [10]. Animals were mechanically ventilated (Ventilator 711^®^; Siemens AG, Munich, Germany); a volume-controlled mode was used with continuous positive pressure ventilation and positive end-expiratory pressure of 5 cm H_2_O, tidal volume and respiratory frequency were adjusted to maintain the peak inspiratory pressure below 30 mm Hg, and an inspiratory oxygen fraction of 0.3 and an inspiratory/expiratory ratio of 1:2 were used. The core body temperature was kept within the normal ranges of young pigs (38°C to 39°C) by means of a warm touch (Tyco Healthcare Deutschland GmbH, Neustadt/Donau, Germany).

#### Instrumentation

We used a combination of the cutdown procedure and Seldinger's technique to expose blood vessels and introduce the following catheters: An 8.5-French sheath and a pulmonary artery catheter (CritiCath™, SP5127 S-TIP TD; Becton Dickinson GmbH, Heidelberg, Germany) were inserted into the right jugular vein. An axial dual-lumen catheter (11 French, 20 mm long, side holes; Gambro Dialysatoren GmbH) was introduced into the right femoral vein. The left femoral vein served for alternative vascular access with an 8.5-French sheath (Arrow Deutschland GmbH) (Alt Cath) for blood delivery in case of insufficient Qb via the arterial line of the dual-lumen catheter. Rinsing of catheters was performed with normal saline and without any anticoagulation.

#### Application of colloids and crystalloids

After BS values were measured, pigs received a bolus infusion of 14 ml/kg of the respective rheologically relevant volume substitute. CVVH was started, and after a period of 10 to 15 minutes for CVVH equilibration, 'CVVH' baseline values were measured. Consecutively, a pump-controlled infusion of the rheologically relevant specific volume substitute was maintained (3.9 ml/kg per hour) beside a basic crystalloid infusion of 5.1 to 5.4 ml/kg per hour, which was suitable for keeping the animals' mean arterial blood pressure above 50 mm Hg.

#### Hemodynamics

A Hewlett-Packard monitor (HP 66S; Hewlett-Packard Development Company, L.P., Bad Homburg, Germany) was used for hemodynamic measurements. CVP was continuously measured and recorded every 30 minutes. CO was measured by thermodilution using 5-ml bolus injections of normal saline at room temperature (mean of five consecutive measurements) every 60 minutes. Arterial pressure was monitored via a femoral artery catheter.

#### Hemorheological and hemocompatibility parameters

Platelet counts, white blood cell (WBC) counts, hematocrit (Hct), free hemoglobin (fHb), plasma ALB, total protein (TP), fibrinogen (Fib), and ALB/TP ratio were determined at the local Institute for Clinical Chemistry (Charité, Universitätsmedizin Berlin, Berlin, Germany). Colloid osmotic pressure (COP) was analyzed from heparinized blood samples by means of a membrane oncometer (BMT 921; Thomae GmbH, Biberach, Germany) (membrane cutoff was 20,000 Da). All parameters were determined at BS, after CVVH equilibration, and after four and seven hours of CVVH. Adjustment of heparinization was based on activated clotting time (ACT), which was determined hourly (or more frequently if required) by means of a Hemochron 400^® ^(ITC, Edison, NJ, USA). Qb rates, catheter-related pressures, and hemodynamic measurements were also determined at these time points and served for the following correlation analyses.

#### Continuous venovenous hemofiltration

An initial heparin bolus of 100 IU/kg BW was followed by a continuous heparin infusion to keep ACT values between 200 and 250 seconds (unfractionated heparin: Liquemin^®^; Hoffmann-La Roche AG, Grenzach-Wyhlen, Germany). CVVH and monitoring of pressures (TMP, Pv, and P_Qa_) were performed using an AK10^® ^machine and corresponding blood and filtration lines made from medical-grade polyvinyl chloride, FH 6S hemofilters (Polyamid S™^®^, membrane surface of 0.6 m^2^, inner diameter of fibers of 215 μm, effective length of 140 mm, and wall thickness of 50 μm). All CVVH materials used were from Gambro Dialysatoren GmbH. CVVH was operated in a closed mode with returning filtrate to the venous bubble trap for five hours; during the last two hours, post-dilution CVVH was operated in a standard open mode, which means that the filtrate was no longer returned for the benefit of crystalloidal volume substitution, which was initiated instead. At the end of the experiments, filters were disconnected from the animals and rinsed with 2 liters of normal saline and a flow rate of 200 ml/minute. Thereafter, filters were cut open, the overall fiber bundle was visually examined, and the percentage of blocked capillaries was estimated by two independent observations from two independent investigators. Blocked fibers were red due to the trapped erythrocytes.

#### Computed tomography scans

To identify the positioning of the catheter tips and blood vessel diameters, two additional animals (30 and 50 kg BW) were scanned after being instrumented with catheter types similar to those used in the experiments. For computed tomography (CT), a 16-channel multi-slice device was used (LightSpeed 16^®^; GE Medical Systems, Milwaukee, IL, USA). The examination protocol for the imaging of the animals consisted of a non-contrast-enhanced scan and a supplementary venous phase-contrast protocol with automatic intravenous injection of 100 ml of non-ionic iodinated contrast media (370 mg/ml iodine). The scan parameters were standardized (tube current of 120 kV and 140 mA, collimated slice thickness of 16 × 1.25 mm, total detector width of 20 mm, rotation speed of 0.5 seconds, and table feed per rotation of 13.75 mm), resulting in a scan speed of approximately 11 seconds for a 30-cm scan length in the z-axis. Image analysis was performed using Advantage Windows 4.2 (GE Medical Systems) and AccuLite (AccuImage Diagnostics Corporation, South San Francisco, CA, USA).

### Statistical analysis

Data were analyzed using Sigma STAT 3.1 and Sigma Plot 8.0 for Windows (Systat Software GmbH, Erkrath, Germany). Because data were not normally distributed, non-parametric tests were used. Inter-group comparisons were performed using the Kruskal-Wallis one-way analysis of variance on ranks followed by pairwise comparison using Dunn's method (that is, for hemorheological subgroups created by different volume substitutes). For inter-group comparison of the two study groups (dual-lumen [group 1] versus Alt Cath [group 2]), the Mann-Whitney *U *test was used. Intra-group analyses comparing start and end of CVVH cycle with continuous Qb were performed using the Wilcoxon rank sum test for paired samples. The Spearman rank order test was used for correlation analyses. Linear regression analysis was performed to determine the dependence of P_Qa _on hemorheological/hemodynamic parameters and the dependence of P_Qa _and Pv on Qb rate. A *p *value of less than 0.05 was considered statistically significant.

## Results

All subgroups with respective volume substitutes provided similar values of BW (in kilograms), hemodynamics, and blood composition tested (data are not shown separated into subgroups). Thus, equal conditions were given for all animals in the two different catheter groups. Regression analysis of BW (in kilograms) versus CO proved dependency of CO on BW (*r *= 0.379; *p *< 0.001). Changes of CO throughout the time course of the protocol within each animal ranged from 0.5 to 2 liters/minute and were independent of the subgroup or the individual BW. Thus, using a range of BW of 35 to 50 kg in immature domestic pigs and a bolus volume load at the beginning was apt to provide clinically relevant ranges of CO (2.48 to 7.53 liters/minute) to verify the hypothesis that CO may be a determinant factor in achievable Qa. Blood composition (that is, low Hct, low COP, and low Fib) showed values of critically ill patients such as after hemorrhage.

### Catheter-related results

**Figure 2:**
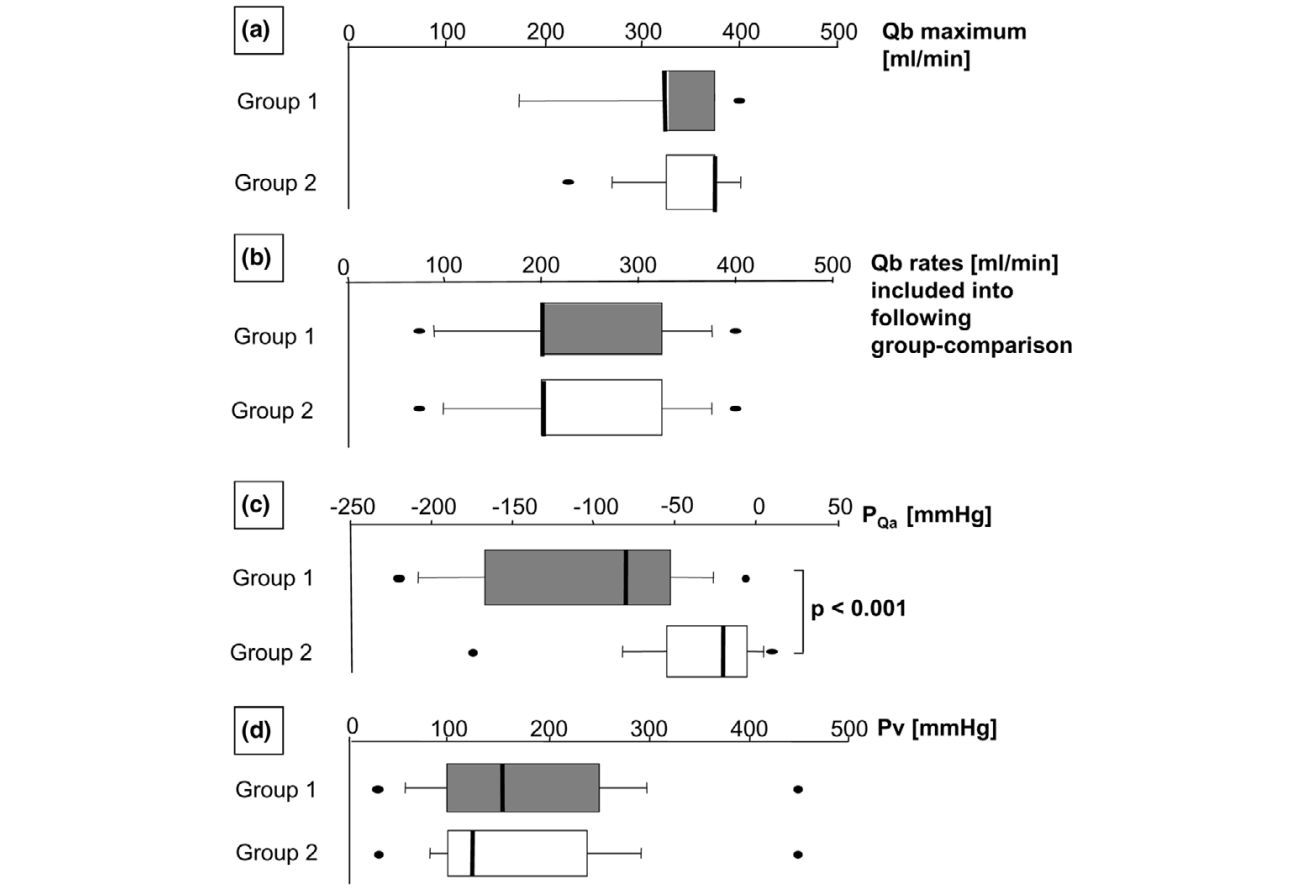
Blood flow (Qb) rates and catheter type-related pressure values for access flow (P_Qa_) and blood return (venous pressure [Pv]). Data of all experiments and time points during continuous venovenous hemofiltration within each study group were summarized, and data distribution is visualized using box plots. The boxes indicate 25th and 75th percentiles, and the medians are shown by the bars within the box plots. Bars provide minimum/maximum values, including outliers. Because we focused on alternatives covering all patients and outliers are part of the very nature of critically ill patients and in turn the topic of this study, we did not accept data classified as outlier. **(a) **Highest Qb rates achievable in the respective study groups. **(b) **A summary of all Qb rates used in the respective study groups. In both catheter study groups, comparable blood flow rates were used with similar distributions of different Qb rates. **(c) **P_Qa _values measured in the respective study groups. **(d) **Pv levels measured in the respective study groups.

As mentioned in the description of the study design, in approximately 44% of the experiments, dual-lumen catheters were not suitable to allow high Qb rates (that is, of more than 300 ml/minute). In three animals, even an initial Qb rate as low as 75 ml/minute could not be achieved and consequently the use of the Alt Cath was necessary. Figure 2a displays the maximal Qb values achieved with the two different catheter types within CVVH system pressure thresholds. In both groups, Qb could have been higher if just the thresholds for P_Qa _had been considered. However, with respect to a limitation for a further increase in Qb, group 2 (Alt Cath) demonstrated wider discrepancies between TMP thresholds and P_Qa _thresholds than the dual-lumen group.

Additional box plots (Figure 2b) demonstrate a similar Qb distribution throughout the time span of the protocol for the two groups. Although there were fewer difficulties in achieving high Qb values in animals handled continuously with the dual-lumen catheter, P_Qa _was significantly lower for group 1 than for group 2 (Alt Cath) (Figure 2c). Interestingly, as indirectly determined by monitoring of the resulting Pv levels, no differences were found for the flow resistance in the venous line of dual-lumen catheters between the groups (Figure 2d).

**Figure 3:**
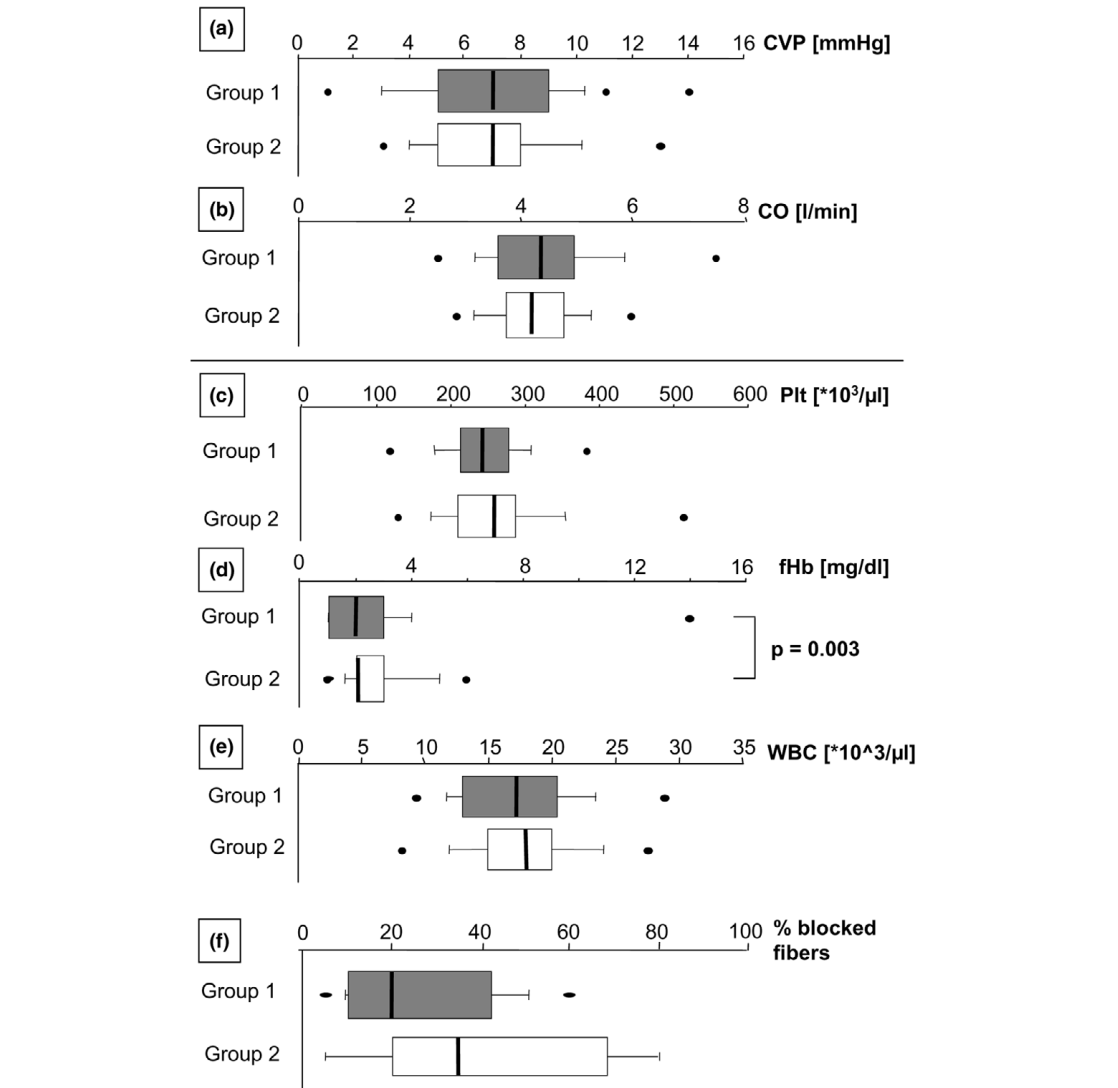
Parameters assumed to influence blood flow (Qb) rates achievable via the blood delivery line of catheters. Data of all experiments and time points during continuous venovenous hemofiltration within each study group were summarized, and data distribution is visualized using box plot presentation. The boxes indicate 25th and 75th percentiles, and the medians are shown by the bars within the boxes. Bars provide minimum/maximum values, including outliers. **(a,b) **Hemodynamic parameters. Central venous pressure (CVP) **(a) **and cardiac output (CO) **(b)**, which are assumed to determine access flow via the arterial line of dual-lumen catheters. **(c-f) **Biocompatibility parameters for the two study groups. **(c) **Plt, platelets. **(d) **fHb, free plasma hemoglobin. **(e) **WBC, white blood cell counts. **(f) **Percentage of finally blocked fibers in the hemofilter.

There was no statistically significant impact of any particular volume substitute on the functionality of the catheters (that is, P_Qa_, Pv, and Qb maximum). Hemorheologically important data describing the blood composition, such as COP and TP (for blood viscosity), ALB/TP ratio (for blood cell aggregability), Fib values (blood cell aggregability, coagulation, and viscosity), WBC (fluid dynamics, cell adhesion, and clot formation), and Hct (cell aggregability and viscosity), did not significantly differ between the two main study groups (therefore, data are not shown). Likewise, no regression of P_Qa _with any of the aforementioned, hemorheologically relevant, potential trigger parameters was found, although these parameters were within critical ranges for hemorheology in critically ill patients. However, CO and CVP did not differ between the groups or influence the catheters' functionality (Figure 3a,b). There was no linear regression for P_Qa _and CO or CVP.

### Biocompatibility of catheters

Given that the catheters were not the only artificial devices that could contribute to adverse side effects in biocompatibility in the present study, the following results were found. Although rinsing of catheters was performed with pure saline without heparinization, no clot formation at the catheter tips was observed in group 2 (Alt Cath) at the end of the experiment. However, some Alt Caths had thin blood cell layers on the inner surface close to the hemostasis valve but no distinct clot formation in that area. Dual-lumen catheters of group 1 demonstrated more or less pronounced clot formation at the side holes for blood delivery. The latter indicated side hole-associated sensitivity for rinsing procedures without anticoagulation. We found clot formation most often at the tip for blood return, which is in confirmation with the results for Pv. Blood values for platelet and WBC counts (Figure 3c,e) did not differ between the catheter study groups. fHb (Figure 3d) was significantly higher in group 2 (Alt Cath), in which additionally a tendency (not significant) for a higher percentage of blocked hemofilter hollow fibers was observed (Figure 3f), but fHb remained at the lower limits of normal ranges and the protocol did not allow us to discriminate between catheter- and hemofilter-related hemolysis.

**Figure 4:**
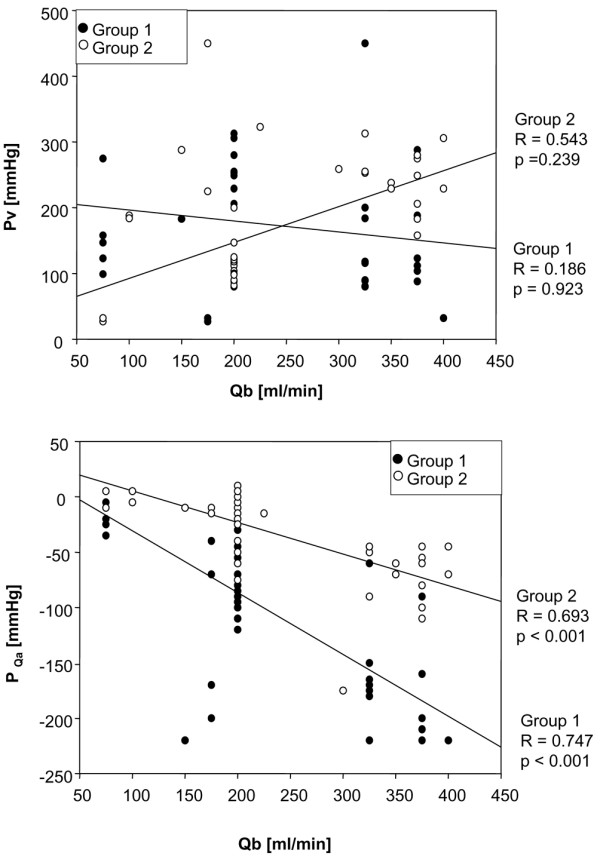
Linear regression analysis for the dependency of pressure of access flow (P_Qa_) and venous pressure (Pv) on blood flow (Qb). All data obtained during continuous venovenous hemofiltration within each study group were summarized for regression analysis independent of the time point or the protocol. For the linear regression analysis of the dependency of Pv on Qb, it has to be considered that the venous line of the dual-lumen catheter was used for blood return in both catheter groups throughout the whole time course of the protocol. However, there was an important difference for Pv level development between the groups. In the alternative catheter group, access flow (Qa) was performed via a contralateral vein as compared to blood return, whereas in the dual-lumen group, only a specific distance between the holes for blood return and the holes for blood delivery separated flow dynamics at the catheter tip for Qa from blood return.

However, a significant linear regression for Qb and corresponding P_Qa _was found for both catheter types, although the regression was higher for the small and long lumen in the dual-lumen catheter (group 1) than for the Alt Cath (group 2) (Figure 4). Interestingly, the venous line, which is the positive pressure section of the CVVH system, did not show linear regression of Qb and flow resistance (Pv) (Figure 4) in either group.

**Figure 5:**
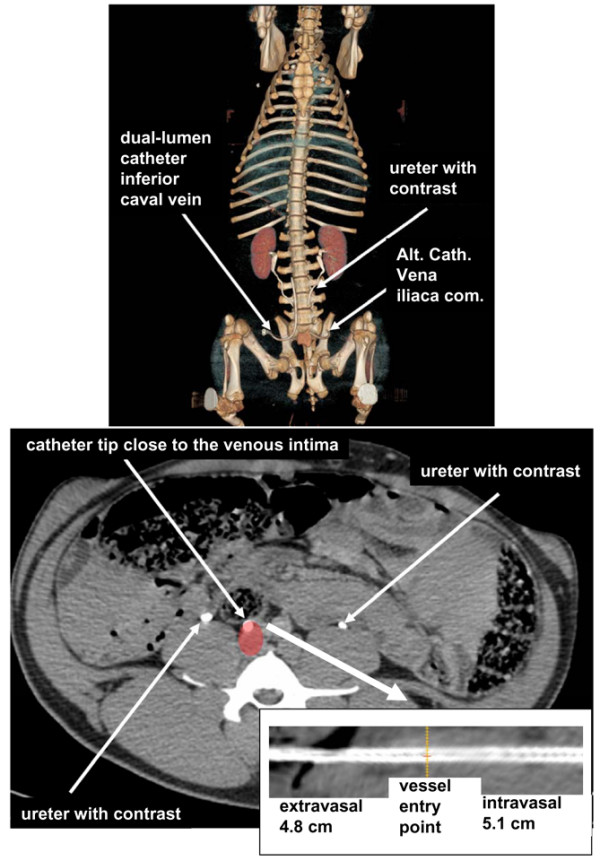
Computed tomography (CT) scans for identification of catheter tip position (body weight of 50 kg). All images are derived from a pig that was treated with intravenous contrast media. Although the time span between application and CT scan allowed contrasting ureters, contrast in the vascular system did not pronounce the veins. The upper image shows three-dimensional reconstructed volume. Due to rapid renal excretion of the contrast media (ureters are contrasted), vessels are not visible. Both catheters (dual-lumen and alternative catheter [Alt Cath]) enter the vessels at the level of the pelvis. Whereas the tip of the dual-lumen catheter is positioned in the inferior caval vein, the tip of the Alt Cath is positioned in the common iliac vein. The lower images are derived from non-contrast-enhanced imaging of the pelvis in the 'abdomen window' (window 350, center 50 Hounsfield units). In the area of the inferior caval vein (red), the dual-lumen catheter tip was close to the inner wall of the vein. Curved reconstruction was performed for the Alt Cath. Orange line shows the vessel entry point. Inferior caval veins were 11 × 17.6 mm in diameter.

**Figure 6:**
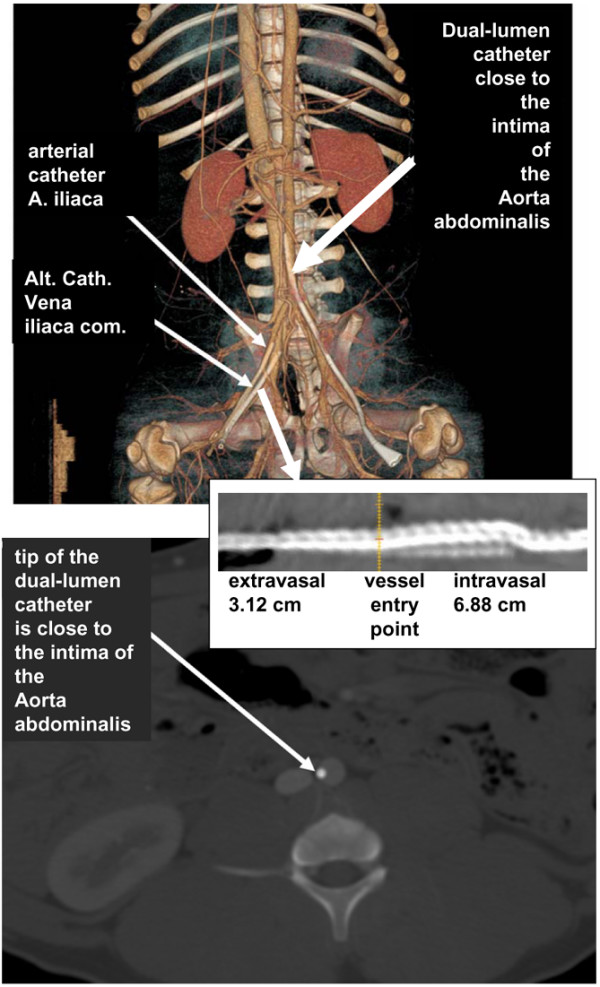
Computed tomography scans for identification of catheter tip position (body weight of 30 kg). Diameters of blood vessels are 10.88 × 9.33 mm (aorta), 6.6 × 9.3 mm (common iliac vein), and 13.6 × 8.86 mm (inferior caval vein at the level of the dual-lumen catheter tip). The upper image shows a three-dimensional reconstruction of the pig from a mixed arteriovenous, contrast-enhanced scan. With respect to all three catheters, the tip was close to the vessel wall. Contrast-enhanced image of the pelvis in the bone window (window 2,500, center 500 Hounsfield units [HU]) to visualize the catheter in contrast-filled aorta. In this pig, the dual-lumen catheter was inserted into the arterial system in order to determine whether the tip position of the dual-lumen catheter at the intima depends on flow dynamics and vessel wall characteristics. The lower image shows an enhanced image of the pelvis in the bone window (window 2500, center 500 HU) to visualize the catheters in contrast-filled vessels. In this animal, curved reconstruction was performed for the alternative catheter (Alt. Cath.). Orange line shows the vessel entry point. The catheters are visualized lying in the right and left iliac arteries and the right iliac vein. Interestingly, the dual-lumen catheter again was visualized at the lateral wall of the blood vessel.

The CT scans indicate that the catheter tip of the 8.5-French sheath (Alt Cath) did not reach the vena cava inferior but was positioned in the common iliac vein (Figures 5 and 6). Thus, differences in regional CO values had no impact on Qa via the respective catheter. Furthermore, images of dual-lumen catheters showed that the tip of the catheter was decentralized and was located close to the intima of the vein (Figure 5). Because low CVP or smaller veins with thinner walls could be assumed to be a reason for this observation, we also investigated the resulting positioning of the catheter tip in the artery system, which is the strongest contrast of basic conditions for a catheter positioning. As demonstrated in Figure 6, introduction of an 11-French, 20 cm-long, dual-lumen catheter into the artery system via the femoral artery results in an intima-close position of the dual-lumen catheter tip. Thus, contact of the tip holes to the blood vessel intima is not a vein-specific feature. Taken together with the results of blood vessel diameters and vessel type in which the catheters were positioned, neither vessel diameter, nor regional CO, nor Qb dynamics could be identified as a potential trigger for Qa during CVVH in anesthetized, ventilated pigs.

## Discussion

This study was performed to verify reasons for limited Qa via dual-lumen catheters when placed in central veins (vena cava via puncture of the femoral vein). Our main results are as follows: (a) The 8.5-French Alt Cath with tip position in the common iliac vein provided higher flow rates than an axial 11-French dual-lumen catheter with side holes in the caval vein and achieved flow rates described for 14.5-French catheters with right atrial tip position [2]. (b) Neither CO, CVP, blood vessel diameters, vessel type nor differences in hemorheology based on different types of volume substitutes were proven to correlate with the levels of negative pressure arising from the arterial line of the dual-lumen catheter or the Alt Cath. (c) Qb rate correlated with the flow resistance (P_Qa_) for Qa but did not correlate with flow resistance for blood return (Pv levels).

Although the idea that available Qa rates via central venous approach often depend on hemodynamics in the critically ill appears to be quite reasonable, we could not confirm this assumption in our porcine model based on femoral vein access with an axial, side-hole, 11-French, dual-lumen catheter with tip position in the caval vein. In agreement with the functional *intra vitam *results of our study, catheter position was not the main determinant for Qa in the way previously assumed. Qa was found to be independent of low or high CO and CVP values as well as of hemorheological differences as far as could be determined in this study. Because we aimed at the highest Qb and filtration rates, we had to increase and reduce flow rates stepwise according to pressure thresholds, including the use of a filtration pump to generate equal net filtration rates comparably between the study groups. Therefore, we could not use the hemopermeability index (spontaneous ultrafiltration rate divided by TMP), which would have been helpful to indicate membrane fouling/clogging during the time course of the protocol and in turn could have indirectly indicated differences in hemorheology [11]. The use of long dual-lumen catheter aims at positioning the catheter tip in the caval vein and thereby providing the highest regional CO values, largest diameter, and highest regional volume state. The best way to achieve these conditions is to place the catheter tip into the right atrium, providing blood delivery and return via a single-vein approach [2].

The CT scans of instrumented animals show that the catheter tip position (common iliac vein) of the Alt Cath used in the present study can be assumed to be insufficient for high Qa. Nonetheless, the Alt Cath provided higher Qa than the axial, side-hole, dual-lumen catheter in the caval vein position. The flow rates achieved were similar to those described for the right atrial approach with 14.5-French catheters [2]. Again, one has to consider that in the animals handled with the Alt Cath, the dual-lumen catheter failed to deliver sufficient Qa. After we switched to the Alt Cath in these animals, only the hemofilter or the Pv level limited a further increase in flow rates. Thus, the Alt Cath provided a favorable performance. Because high-volume hemofiltration meanwhile becomes of greater interest for therapy of sepsis and multiple organ failure, our results may be quite helpful for overcoming low-flow problems from the side of vascular access in many patients. The use of alternative principles such as a 'dual-vein approach' could also reduce the filter clearance limitations due to high Pv levels as investigated and recently published elsewhere [8,12].

Commonly used catheters for renal replacement therapies, plasmapheresis, hemoperfusion, or extracorporeal membrane oxygenation are introduced via Seldinger's technique to reduce maneuver-related trauma. Therefore, these catheters require mechanically stable, cone-forming catheter tips to facilitate the percutaneous insertion through the connective tissue without any deformation. The tapered catheter tip is another limiting factor for flow dynamics/resistance [13]. Although side holes may provide higher Qa rates in the case of right atrial position, they also increase the risk of clot formation and infections. Placed in a central vein position, side holes may lead to intima attachment, reducing Qa and increasing the risk of thrombus formation. Thus, by the introduction of a modified Seldinger's technique whereby the function of a tapered tip is transferred to an introducer, catheters with a wide circular tip hole could be beneficial with respect to blood delivery.

## Methods

The use of immature pigs provides the unfavorable hemorheological conditions often encountered in critically ill patients due to multiple etiologies [14-16]. In pigs, however, unfavorable hemorheological conditions occur due to the pigs' physiology and not in response to pathophysiological conditions. Furthermore, the CO values in pigs were within the ranges relevant in critically ill patients.

However, experimental conditions are not comparable to the clinical situation. We had to operate CVVH with a return of the filtrate to the animal for several hours in order to avoid a severe washout of healthy animals. As a result, washout was restricted to the last two hours in the open post-dilution mode. We integrated a small hemofilter into the setting because the hemocompatibility effects of all CVVH components mutually affect each other and thus have to be considered for clinical relevance of results. Furthermore, this setting allowed us evaluate whether a small hemofilter (0.6 m^2 ^effective membrane surface) or the respective catheter type was the limiting aspect for a potential increase of CVVH-related clearance based on post-dilution mode. We chose an axial dual-lumen catheter with side holes for blood delivery and return because this type was declared by two companies to be the type most often sold (and thus used) for CVVH. Because we chose a small hemofilter for small overall surface of the extracorporeal system and young pigs, we also used a smaller size (11 French). In addition to the failure of the dual-lumen catheter concerning Qa, the Pv was the major limit for filtration performance by increasing the TMP above a tolerable threshold, which is in confirmation with our former *in vitro *study [8]. Thus, also from a retrospective point of view, running a complete CVVH appears to be the best choice for our study design. Because there were no clear signs of hemo-incompatibility, the 8.5-French sheath used in this setting was also adequate for providing sufficient blood delivery via a femoral access. Nonetheless, with respect to appropriate catheter engineering, there will be better catheter alternatives for clinical approach of the 'dual-vein principle' whenever right internal jugular vein access is not possible.

One limitation of this study is that all analyses were based on the flow rate value set by the pump. However, given that a reduction of Qb in the case of very low, negative pressures for Qa was already demonstrated by others [1], one could assume that the Qb rates in the dual-lumen group in particular often did not reach flow rates set by the pump. Therefore, the tendency for superiority in Qa for the Alt Cath group would probably become highly significant if data analysis could have been based on real Qb measurements. Another limitation is the lack of randomized allocation of animals to the catheter groups. On the other hand, our setting provided an intra-individual control between the catheter types. This 'crossover' situation is the only setting possible for proving that switching to an alternative vascular access may solve low-flow problems based on established approaches in the respective animal or patient.

## Conclusion

In this study on CVVH in healthy pigs, we found that blood delivery rates from the caval vein via a femoral vein access and thus possibly filter clearance were not correlated with hemodynamics and hemorheology but depended highly on the flow resistance given by the arterial line of the femoral vein catheter. When a percutaneous modified Seldinger's technique with an introducer/sheath combination was used, even a more distal venous catheter position provided flow rates exceeding those described for 14.5-French dual-lumen catheters with right atrial tip position in humans. Because the experimental model was set to accelerate unfavorable effects in biocompatibility, a direct comparison to clinical circumstances is not given. However, if the right internal jugular vein/atrium approach is hampered in the critically ill patient and the usual maneuvers to increase filtration performance (such as pre-dilution, heparin priming, and anticoagulatory catheter locks) do not work, a 'dual-vein approach' could be the last option for a high clearance in CVVH without further upscaling of hemofilters. Thus, it would be of interest to obtain clinical data to validate these first experimental results.

## Key messages

• Blood delivery rates from the caval vein via an axial, side-hole, dual-lumen catheter did not correlate with hemodynamics, size of blood vessels, or hemorheological impact of colloids.

• Even with low CO, a short 8.5-French catheter with a central tip hole provided Qb from the common iliac vein comparable to long 14.5-French dual-lumen catheters with right atrial tip position.

• It seems worthwhile to clinically re-investigate the principle of 'dual-vein approach' with large vein access for blood delivery and probably peripheral vein access for blood return in order to provide alternatives when established approaches are failing.

## Abbreviations

ACT = activated clotting time; ALB = albumin; Alt Cath = alternative catheter; BS = 'native' baseline; BW = body weight; CO = cardiac output; COP = colloid osmotic pressure; CT = computed tomography; CVP = central venous pressure; CVVH = continuous venovenous hemofiltration; fHb = free hemoglobin; Fib = fibrinogen; Hct = hematocrit; P_Qa _= pressure of access flow; Pv = venous pressure; Qa = access flow; Qb = blood flow; TMP = transmembrane pressure; TP = total protein; WBC = white blood cell.

## Competing interests

This study was supported in part by Fresenius Kabi AG and Gambro Dialysatoren GmbH. The CT scans were supported by Ellegaard Göttingen Minipigs ApS (Dalmose, Denmark) and Raumedic AG (Münchberg, Germany). However, since none of these companies develops or sells its own catheter products for vascular access in extracorporeal treatments, competing interests are unlikely for either the authors or the companies.

## Authors' contributions

JKU was head of the working group; she performed the experiments, wrote the paper, and acquired all of the funding for this study. SMN and A-JL performed CT scans and detailed analyses of catheter positioning and blood vessels and participated in drafting the manuscript. KP, RCF, MMT, and JB all significantly participated in instrumentation, CT experiments, discussion and interpretation of the results, design of the study, and draft of the manuscript and provided the clinical background for all aspects of intensive care medicine. MMT had significant impact on the discussion of methods in porcine models and hemolysis in pigs undergoing extracorporeal treatments. All authors have read and edited the manuscript, and the final, submitted version is approved by all of them.
